# Immunobiological signatures and the emerging role of SPP1 in predicting tumor heterogeneity, malignancy, and clinical outcomes in stomach adenocarcinoma

**DOI:** 10.18632/aging.205148

**Published:** 2023-10-26

**Authors:** Yanan Wu, Lingyu Ren, Yichun Tang, Zhu Zhu, Shifan Liu, Yan Jiang, Siming Zhang, Xiaocan Zhuang, Yuanbiao Chen

**Affiliations:** 1 Department of Gastroenterology, Rudong People’s Hospital, Rudong Hospital Affiliated to Nantong University, Nantong, China; 2 Cancer Research Center Nantong, Nantong Tumor Hospital and Affiliated Tumor Hospital of Nantong University, Nantong, China; 3 Department of Medical Imaging, Medical School of Nantong University, Nantong, China; 4 Department of Engineering Training Center, Nantong University, Nantong, China; 5 Affiliated Hospital of Youjiang Medical University for Nationalities, Baise, China

**Keywords:** stomach adenocarcinoma, tumor microenvironment, immunotherapy, multi-omics, SPP1

## Abstract

Background: Immunotherapy, as a form of immunobiological therapy, represents a promising approach for enhancing patients’ immune responses. This work aims to present innovative ideas and insights for prognostic assessment and clinical treatment of stomach adenocarcinoma (STAD) by leveraging immunobiological signatures.

Methods: We employed weighted gene co-expression network analysis (WGCNA) and unsupervised clustering analysis to identify hub genes. These hub genes were utilized to construct a prognostic risk model, and their impact on the tumor microenvironment (TME) and DNA variations was assessed using large-scale STAD patient cohorts. Additionally, we conducted transfection experiments with plasmids to investigate the influence of SPP1 on the malignancy of HGC27 and NCI-N87 cells.

Results: Unsupervised clustering of 12 immune-related genes (IRGs) revealed three distinct alteration patterns with unique molecular phenotypes, clinicopathological characteristics, prognosis, and TME features. Using LASSO and multivariate Cox regression analyses, we identified three hub genes (*MMP12, SPP1, PLAU*) from the IRGs to establish a risk signature. This IRG-related risk model significantly stratified the prognosis risk among STAD patients in the training (*n* = 522), testing (*n* = 521), and validation (*n* = 300) cohorts. Notably, there were discernible differences in therapy responses and TME characteristics, such as tumor purity and lymphocyte infiltration, between the risk model groups. Subsequently, a nomogram that incorporates the IRG signature and clinicopathological factors demonstrated superior sensitivity and specificity in predicting outcomes for STAD patients. Furthermore, down-regulation of SPP1, as observed after siRNA transfection, significantly inhibited the proliferation and migration abilities of HGC27 and NCI-N87 cells.

Conclusions: In summary, this study highlights the critical role of immune-related signatures in STAD and offers novel insights into prognosis indicators and immunotherapeutic targets for this condition. SPP1 emerges as an independent prognostic factor for STAD and appears to regulate STAD progression by influencing the immune microenvironment.

## INTRODUCTION

Stomach adenocarcinoma (STAD) is the most common pathological subtype of gastric cancer (GC), characterized by exceptionally high recurrence and metastasis rates [1]. Research indicates that STAD originates from the gastric mucosal epithelium and ranks fifth among all malignant tumors in terms of incidence [2]. Therefore, it is imperative to employ rigorous scientific standards and methodologies for timely diagnosis, rational treatment, and accurate prognosis prediction.

The tumor microenvironment (TME), comprising tumor cells, immune cells, and stromal cells (e.g., fibroblasts and endothelial cells), plays a pivotal role in cancer development [3, 4]. Activated fibroblasts are key players in the intricate process of tumor-stromal interactions, influencing tumor growth, angiogenesis, and other crucial processes [5]. Mesenchymal stem cells (MSCs), along with their differentiated counterparts, constitute the predominant and critical components of the tumor mesenchyme, significantly impacting the phenotype of immune cells and thereby influencing tumor progression [6]. Mounting evidence suggests that the TME is closely intertwined with predicting immunotherapy responses in STAD [7].

As the most prevalent malignancy within the digestive system, GC has shown resistance to conventional chemoradiotherapy, necessitating the exploration of novel treatment modalities [8]. The elevated incidence of somatic mutations in GC patients makes immunotherapy an appealing therapeutic avenue for gastric cancer [9]. Immunotherapy targets immune cells with higher infiltration levels, aiming to maximize the clinical survival rates of patients [10]. Aberrations in immune checkpoints profoundly affect the development, invasion, and metastasis of advanced STAD. In recent years, immune checkpoint inhibitors (ICIs), notably anti-PD-1/PD-L1 antibodies, have revolutionized traditional treatment paradigms, exhibiting enhanced efficacy in STAD treatment [11]. Furthermore, the immune status within the tumor may significantly impact patient prognosis. However, the precise alterations in gene expression profiles and molecular mechanisms related to tumor immunity remain unclear. In this study, our objective is to offer fresh insights into the prognosis evaluation and clinical management of STAD by exploring risk characteristics and constructing an effective signature centered on immune-related genes (IRGs).

In this investigation, we have modularized IRGs based on their expression levels and subsequently identified 12 genes through correlation and univariate Cox analysis. Our unsupervised clustering analysis has unveiled three distinct clusters, with an overlapping set of four genes. Utilizing the least absolute shrinkage and selection operator (LASSO) and multivariate Cox analysis, we have identified three hub genes, which form the foundation of our prognostic risk signature. Subsequently, we delve into further studies on the TME, immunotherapy, and patient prognosis.

## MATERIALS AND METHODS

### Data acquisition and processing

The experimental data and clinical annotation with public access come from the Gene Expression Omnibus (GEO; https://www.ncbi.nlm.nih.gov/geo/) and the Cancer Genome Atlas (TCGA; https://portal.gdc.cancer.gov/) databases. Patients lacking survival information are excluded from further analysis. For subsequent analysis, clinical and transcriptomic data for four GEO cohorts (GSE15459, GSE34942, GSE38749, and GSE84437), as well as a TCGA-STAD cohort, are obtained. To characterize the transcriptomic profiles of the tumor immune microenvironment landscape, we included a total of 2660 immune-related genes (IRGs) sourced from https://www.immport.org/home. Subsequently, we converted Fragments Per Kilobase Million (FPKM) values into Transcripts Per Kilobase Million (TPM) and merged the four datasets while addressing batch effects across multiple samples.

### Construction of weighted gene co-expression network analysis (WGCNA)

We employed the standard WGCNA procedure to analyze the co-expression network of the 2660 immune-related genes (IRGs) to identify STAD genes with strong correlations to immune cells. Utilizing the WGCNA package (http://www.r-project.org/), we imported and processed the transcriptome data, filtering out genes with no significant differences between groups. The gene expression data of TCGA-STAD patients were closely examined to identify potential markers associated with IRG characteristics in patients.

Within the context of a scale-free co-expression network, we removed overly divergent samples by pruning samples with a Height > 75 and adhering to the Scale Free Topology signatureFit criterion, ensuring an R2 > 0.9. Subsequently, we transformed the expression matrix into an adjacency matrix and further into a topology matrix. Genes were clustered based on the Topological Overlap Matrix (TOM) using mean linkage hierarchical clustering, with each resulting module containing a minimum of 30 genes. We identified gene modules using dynamic shearing methods and isolated characteristic genes within each module for cluster analysis. After merging modules with a similarity >0.25, we conducted an in-depth analysis of the newly formed module and assessed the association of eigenvectors with STAD prognostic outcomes.

### Unsupervised clustering analysis of prognostic immune-related genes

Correlation and univariate Cox analysis were conducted on the 60 genes identified within the black module. Out of these, 12 genes exhibited *p*-values less than 0.05, and these were selected for further analysis (Supplementary Table 1). We then utilized the STRING website (https://string-db.org/) to identify proteins associated with these 12 genes and visualized the interactions using Cytoscape software. Subsequently, employing unsupervised clustering analysis, we delineated distinct clusters based on the enrichment of STAD-related genes.

### Exploring the clinical relevance and enrichment analysis of molecular subtypes

Our study delves into the relationship between the three clusters resulting from the unsupervised clustering of the 12 genes, clinicopathological characteristics, and prognosis. Various patient characteristics were analyzed, including age, gender, race, tumor status, tumor node metastasis (TNM) stage, tumor stage, tumor grade, primary treatment outcome, and future prognosis. We generated Kaplan-Meier curves to assess overall survival (OS) among the three clusters, employing the “survival” and “survminer” R packages.

To gain insight into the distinct characteristics of STAD-related genes in biological processes, we performed Gene Set Variation Analysis (GSVA) on these 12 genes. We used a marker gene set (c2.cp.kegg.v7.4) sourced from the MSigDB database, identifying four genes common to all three clusters. Subsequently, the clusterProfiler package was employed to conduct Disease Ontology (DO) analyses, Kyoto Encyclopedia of Genes and Genomes (KEGG), and Gene Ontology (GO) enrichment analyses on these four genes. Our aim was to elucidate their enrichment patterns, associated pathways, and implications in various disease types within different biological processes, cellular components, and molecular functions.

### Construction and validation of the three hub gene risk signature

For the reason that a single tumor’s genetic characteristics were quantified by the construction of a new risk signature score, we performed LASSO analysis based on the 4 genes obtained above. Subsequently, multivariate Cox regression analysis is performed to construct risk signature in the training set. The “caret” package and samples from the TCGA, GSE15459, GSE34942, GSE38749 and GSE84437 cohorts are randomly split with a ratio of 1:1 into training (*n* = 522) and testing sets (*n* = 521) to construct risk signatures. The risk characteristics are defined as follows:


Risk Score=∑(Expi×Coef)

where the expression of each gene and risk coefficient are represented by Expi and Coefi, respectively. Patients in the training, testing, and all sets are categorized into low-risk and high-risk score groups based on median score. Each group conducts a corresponding Kaplan-Meier survival analysis, and further creates receiver operating characteristic (ROC) curves.

### Correlation between risk signatures and immune cells, pathways, and immune scoring

To ensure the accuracy of our findings, we utilized CIBERSORT with Monte Carlo sampling to calculate empirical *p*-values for deconvolution. Employing CIBERSORT, we explored the relationship between high- and low-risk groups and their respective immune cell compositions. Additionally, we determined the ratio of stromal cells to immune cells using the “estimate” package, leading to the computation of stromal scores, immune scores, estimate scores, and ultimately tumor purity. We also investigated the association of the tumor microenvironment (TME) score with the aforementioned risk groups using the “vioplot” package. Furthermore, data gathered from the TCIA website allowed us to evaluate the therapeutic efficacy of Immune Checkpoint Inhibitors (ICIs) based on Immunophenotype Scores (IPS).

### Correlation of mutations, signatures in STAD, GSVA, and drug sensitivity analysis

We calculated the Tumor Mutational Burden (TMB) by integrating mutation information and phenotype data from each sample. This enabled us to generate box plots depicting TMB distribution across different groups and waterfall plots showcasing the top 20 genes with the highest mutation frequencies, all in conjunction with sample grouping. Furthermore, we conducted microsatellite instability (MSI) analysis using the MANTIS algorithm. This facilitated the calculation of MSI scores for each sample. We then performed Spearman correlation tests to assess associations and obtain *p*-values.

Differential analysis of tumor mutation gene expression data was carried out using the “limma” package in R. Differences with a False Discovery Rate (FDR) < 0.05 and an absolute log2 Fold Change greater than 2 were considered statistically significant. To identify cancer stem cell-related subtypes based on the distinctions in cancer stem cell-related genes, we utilized Principal Component Analysis (PCA) for consistent cluster analysis of STAD samples. Subsequently, we analyzed the proportion of each cancer stem cell-related subtype within the high- and low-risk groups. To verify the stability of our signature, we conducted external validation using the GSE62254 dataset to confirm the survival rates of MMP12, SPP1, and PLAU.

### Establishment and validation of a nomogram scoring system

For each variable, we displayed *P*-values, Hazard Ratios (HRs), and 95% Confidence Intervals (CIs) using the “survival” package. We performed both univariate and multivariate Cox regression analyses to identify relevant terms for constructing nomograms. To determine the nomogram with the highest sensitivity, we generated Receiver Operating Characteristic (ROC) curves for different clinical features, showcasing sensitivity and specificity, along with risk scores for 1-, 3-, and 5-year survival events. Subsequently, we plotted calibration curves for 1-, 3-, and 5-year survival using the “rms” package.

### Cell culture and lentiviral transfection of gastric cancer cell lines

We obtained human gastric cancer cell lines (HGC27 and NCI-N87) from the Cell Bank of Shanghai Institutes of Biological Sciences, Chinese Academy of Sciences (Shanghai, China). These STAD cells were cultured in RPMI 1640 medium (Gibco, CA, USA) supplemented with 10% fetal bovine serum (FBS; Gibco, NY, USA) and 1% penicillin-streptomycin solution (Gibco, NY, USA). The cells were maintained in a humidified incubator with 5% CO_2_ at 37°C. Cells were seeded in 6-well plates and allowed to reach 70% confluency at the time of transfection. Transfections were performed using SPP1-siRNA (General Biol, Taiwan) and overexpression plasmids (GeneChem, Shanghai, China) with Lipofectamine 2000 Transfection Reagent (Invitrogen, ThermoFisher, MA, USA) following the manufacturer’s instructions.

### Real-time quantitative polymerization chain reaction (RT-qPCR)

Total RNA was extracted from HGC27 and NCI-N87 cells using a commercial RNA extraction kit (Beyotime Institute of Biotechnology, Shanghai, China) following the manufacturer’s protocol. Reverse transcription was performed using a cDNA synthesis kit (Takara Bio, Inc., Kusatsu, Japan) with random primers, and qPCR was conducted on a real-time PCR machine with specific primers for the target gene, SPP1. Data analysis involved the 2^−ΔΔCt^ method for relative gene expression, normalizing to GAPDH, and statistical significance was determined using appropriate tests with a significance threshold of *p* < 0.05. The primer sequences (5′-3′) were as follows: SPP1 forward, CTC CAT TGA CTC GAA CGA CTC and reverse, CAG GTC TGC GAA ACT TCT TAG AT; GAPDH forward TGT GGG CAT CAA TGG ATT TGG and reverse, ACA CCA TGT ATT CCG GGT CAA T.

### Western blot analysis

HGC27 and NCI-N87 cells (1 × 10^5^) were plated in 6-well plates. After 24 hours of incubation, cells were treated with DMSO (vehicle), FHP01, or XAV939 (Merck, Darmstadt, Germany) for specified durations. Following transfection, cells were washed with cold PBS, and total protein extracts were obtained by adding 80 μL of RIPA Lysis buffer. Protein samples (10 μg) were loaded onto 8% polyacrylamide gels with 1× Laemmli buffer and resolved by SDS-PAGE. Subsequently, samples were transferred to Immobilon-P PVDF membranes (Millipore, MA, USA, IPVH00010) and probed with Osteopontin Antibody (PA5-32527, Invitrogen, ThermoFisher, MA, USA) and anti-β-actin (15G5A11/E2, Invitrogen, ThermoFisher), as previously described [12].

### Cell counting kit-8 (CCK8) assay

For the CCK8 analysis, 3 × 10^3^ transfected HGC27 and NCI-N87 cells were plated in a 96-well plate with 100 μL of medium. After cell adhesion, supernatants were removed, and serum-free medium containing 10% CCK8 (CCK-8 Kit; Dojindo, Kumamoto, Japan) was added to each well for a 2-hour incubation according to the manufacturer’s protocols [13]. The optical density (OD) values at 450 nm were measured using a microplate reader (BioTek, VT, USA) on days 1, 2, 3, 4, and 5. Each sample was analyzed in triplicate.

### Transwell assay

Transwell assays were performed using Transwell chambers (8-μm pores; Corning Costar, Corning, NY, USA) in 24-well plates. For the cell migration assay, the chambers were coated without matrigel. Cells (1 × 10^5^) in 100 μL of serum-free DMEM medium were seeded in the upper chamber, and 600 μL of DMEM medium containing 20% FBS was added to the lower chamber. The cells were cultured in the incubator for 24 hours. Subsequently, chambers were washed twice with PBS, fixed with paraformaldehyde for 15 minutes, and stained for 10 minutes with a 0.5% crystal violet solution.

### Statistical analysis

Statistical and graphical analyses were carried out using SPSS software (version 25.0) or R software (version 3.4.1). One-way ANOVA tests were used for comparisons among multiple groups (≥3), while unpaired Student’s *t*-tests assessed statistical differences between two groups. All hypothesis testing was two-sided, and a *P*-value of 0.05 or less was considered statistically significant.

### Availability of data and materials

All of software applications and data employed are available from the corresponding authors on reasonable request. GSE15459: (https://www.ncbi.nlm.nih.gov/geo/query/acc.cgi?acc=GSE15459); GSE34942: (https://www.ncbi.nlm.nih.gov/geo/query/acc.cgi?acc=GSE34942); GSE38749: (https://www.ncbi.nlm.nih.gov/geo/query/acc.cgi?acc=GSE38749); GSE84437: (https://www.ncbi.nlm.nih.gov/geo/query/acc.cgi?acc=GSE84437); TCGA: (https://portal.gdc.cancer.gov/).

## RESULTS

### Analysis of WGCNA based on TCGA-STAD cohort

**Figure 1:**
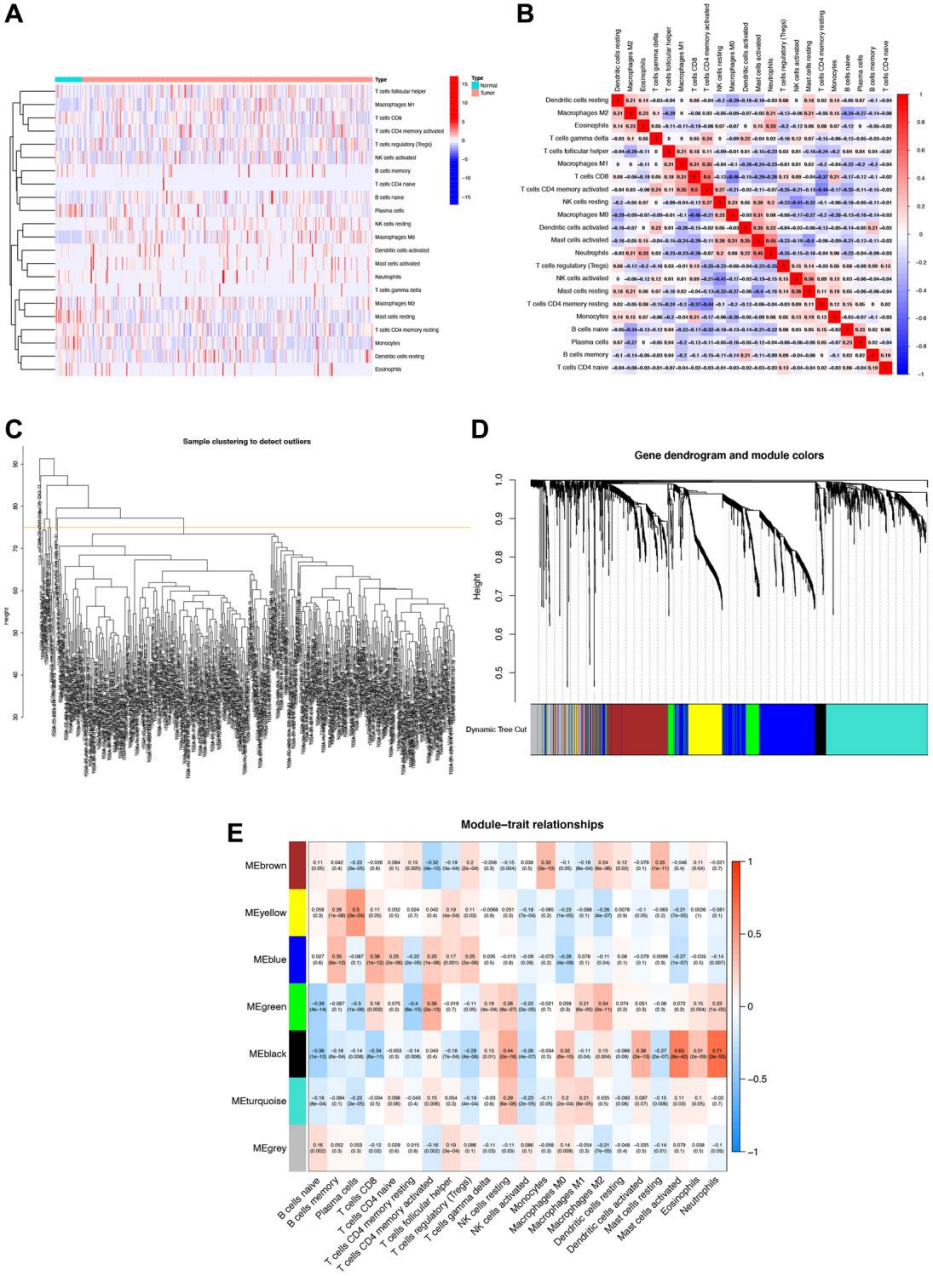
**Immune cell correlation analysis and weighted gene co-expression network analysis (WGCNA).** (**A**) Correlation analysis of immune cell populations in normal and tumor samples, with negative correlations in blue and positive correlations in red. (**B**) Detailed immune cell correlation analysis. (**C**) Sample clustering to detect groups with more than 75 samples. (**D**) WGCNA co-expression analysis showing which module each gene belongs to. (**E**) Clinical correlation analysis of each module to observe the correlation between modules and immune cells, with blue representing negative correlation and red representing positive correlation.

The heatmap clearly demonstrates a strong association between tumor occurrence and immune cells within the TCGA-STAD cohort (Figure 1A, 1B). In constructing the scale-free co-expression network, we removed samples with Height >75, adhering to the Scale Free Topology signatureFit standard with signed R2 >0.9. After analysis, a power value of 5 emerged as the optimal choice (Figure 1C). A gene dendrogram yielded a total of seven modules (Figure 1D). Further examination involved assessing the correlation between eigenvectors and STAD prognostic outcomes within these seven modules. Notably, the black module, containing 60 genes, exhibited the highest correlation with immune cells. Specifically, the genes in this module had the strongest associations with neutrophils (R = 0.71, *P* = 3e-55), activated mast cells (R = 0.63, *p* = 8e-42), and resting NK cells (R = 0.44, *P* = 2e-18) (Figure 1E).

### Correlation, unicox analysis, and functional annotations of immune-related genes (IRGs)

**Figure 2:**
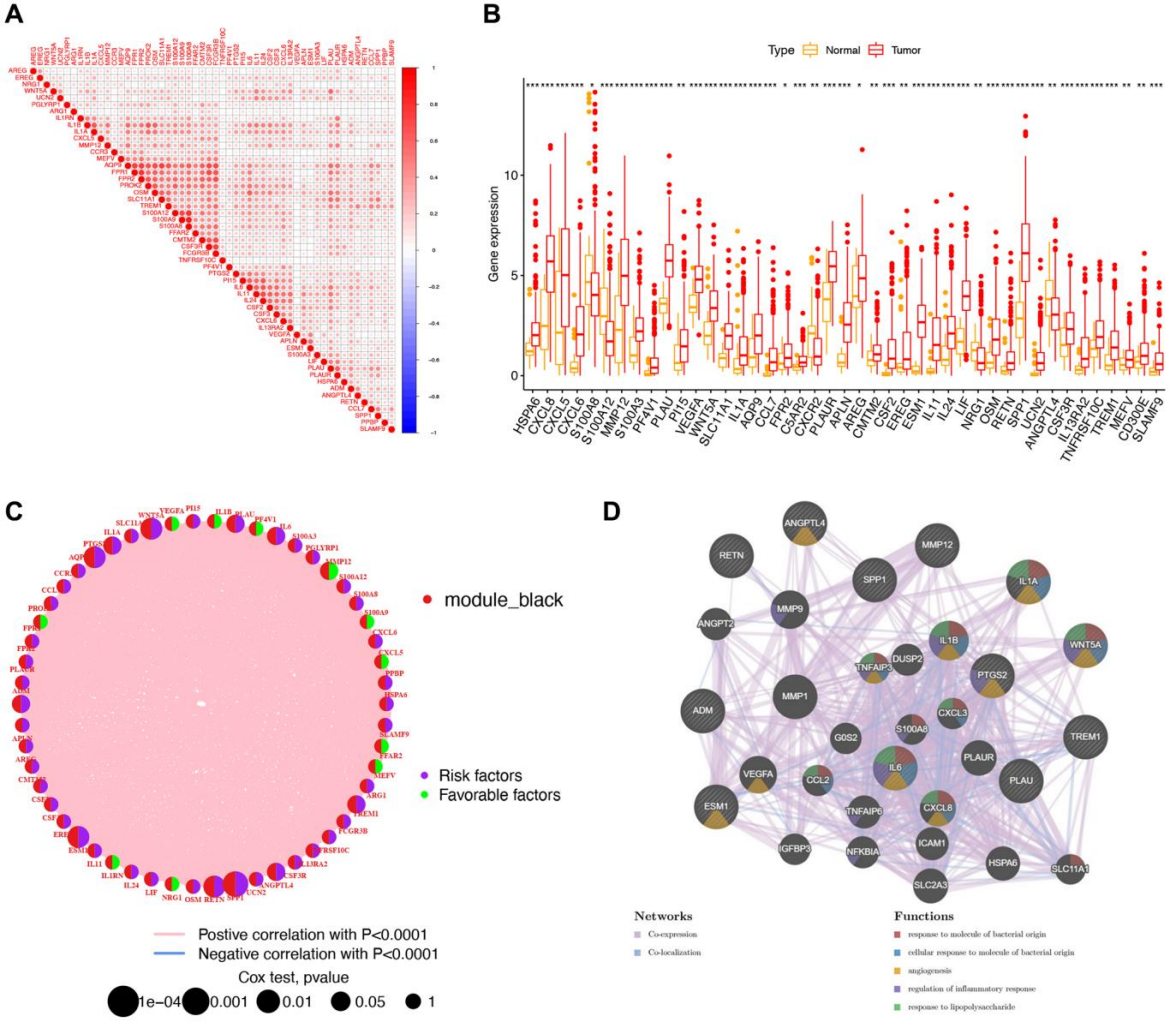
**A study of 60 immune-related genes (IRGs) in stomach adenocarcinoma (STAD).** (**A**) Triangle heat map showing correlation analysis of 60 IRGs. (**B**) Box plot comparing gene expression in normal and tumor groups. ^***^p < 0.001; ^**^p < 0.01; ^*^p < 0.05. (**C**) Network graph, in which nodes represented IRGs, and node size represented the relationship between genes and survival. The left semicircle of the node represented module_black. The green right semicircle showed low-risk genes, and the purple right semicircle showed high-risk genes. (**D**) Visualization of the co-expression network, in which the nodes represent genes, and the size of the nodes represented the number of connected genes.

A triangular heatmap revealed significant correlations at the mRNA level among the 60 screened IRGs (Figure 2A). Additionally, these IRGs exhibited generally high expression levels in tumor samples (Figure 2B). Subsequently, the 60 IRGs underwent unicox analysis, which identified 12 genes with a *p*-value < 0.05 (Figure 2C). A visual analysis of their co-expression relationships and functions indicated that these 12 IRGs were closely associated with molecules of bacterial origin and lipopolysaccharides, played roles in regulating inflammatory responses, and were involved in angiogenesis (Figure 2D).

### Unsupervised clustering based on 12 IRGs

**Figure 3:**
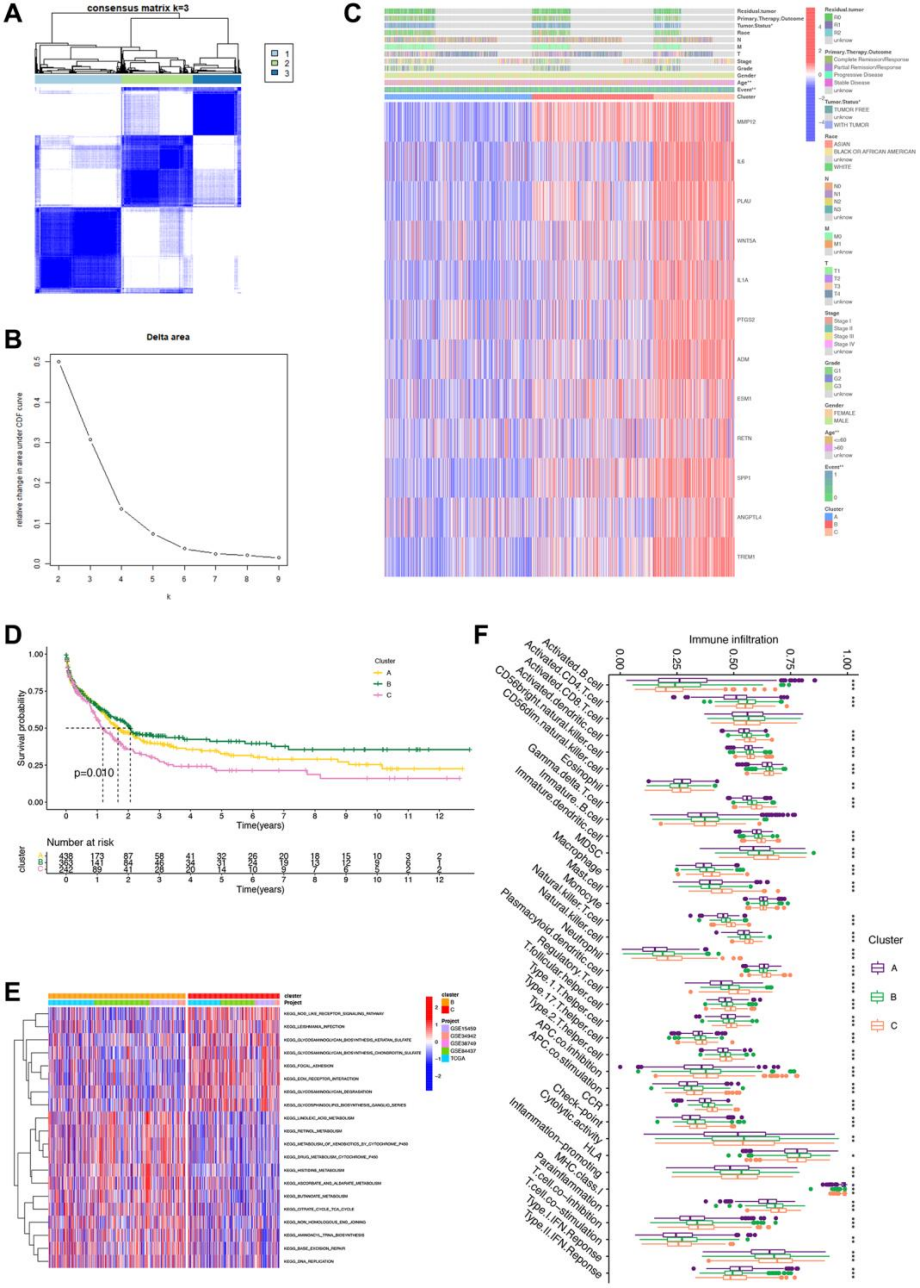
**Unsupervised clustering analysis based on 12 survival genes.** (**A**) Consensus matrix heat map defining three clusters (K = 3). (**B**) Unsupervised clustering analysis. For each k, calculate the relative change in the area under the CDF curve compared to k-1. (**C**) Heatmap based on 12 genes. (**D**) Survival analysis of clusters showing differences in patients’ prognosis between clusters. (**E**) GSVA analysis showing differences in different pathways of different clusters. (**F**) Box plot revealing immune infiltration of different clusters. ^***^p < 0.001; ^**^p < 0.01; ^*^p < 0.05.

Using the expression patterns of the 12 IRGs, we conducted unsupervised clustering across the TCGA, GSE15459, GSE34942, GSE38749, and GSE84437 cohorts to classify STAD samples into distinct molecular subtypes. This analysis revealed three clusters, consisting of 452 samples in the first subtype, 376 samples in the second subtype, and 251 samples in the third subtype (Figure 3A, 3B). The unsupervised clustering across the combined cohort uncovered three distinct alteration patterns with unique molecular and clinical characteristics (Figure 3C). These three clusters were labeled as gene Clusters A, B, and C, respectively. Notably, gene Cluster B exhibited a significant survival advantage compared to the other two gene Clusters, as observed in the prognostic analysis (Figure 3D). Further GSVA enrichment analysis highlighted distinct KEGG pathway enrichment features, with Cluster B showing stronger associations with metabolic pathways and Cluster C with synthetic pathways (Figure 3E). Furthermore, these three gene Clusters displayed distinct immune cell-infiltrating characteristics (Figure 3F). Cluster B exhibited widespread infiltration of immune cells, including activated CD4 T cells and activated CD8 T cells, indicating inflammation-promoting characteristics and high HLA. Conversely, Cluster C displayed an abundance of macrophages, neutrophils, and natural killer cells, signifying parainflammation characteristics.

### GO, KEGG, and DO enrichment analyses of gene clusters

**Figure 4:**
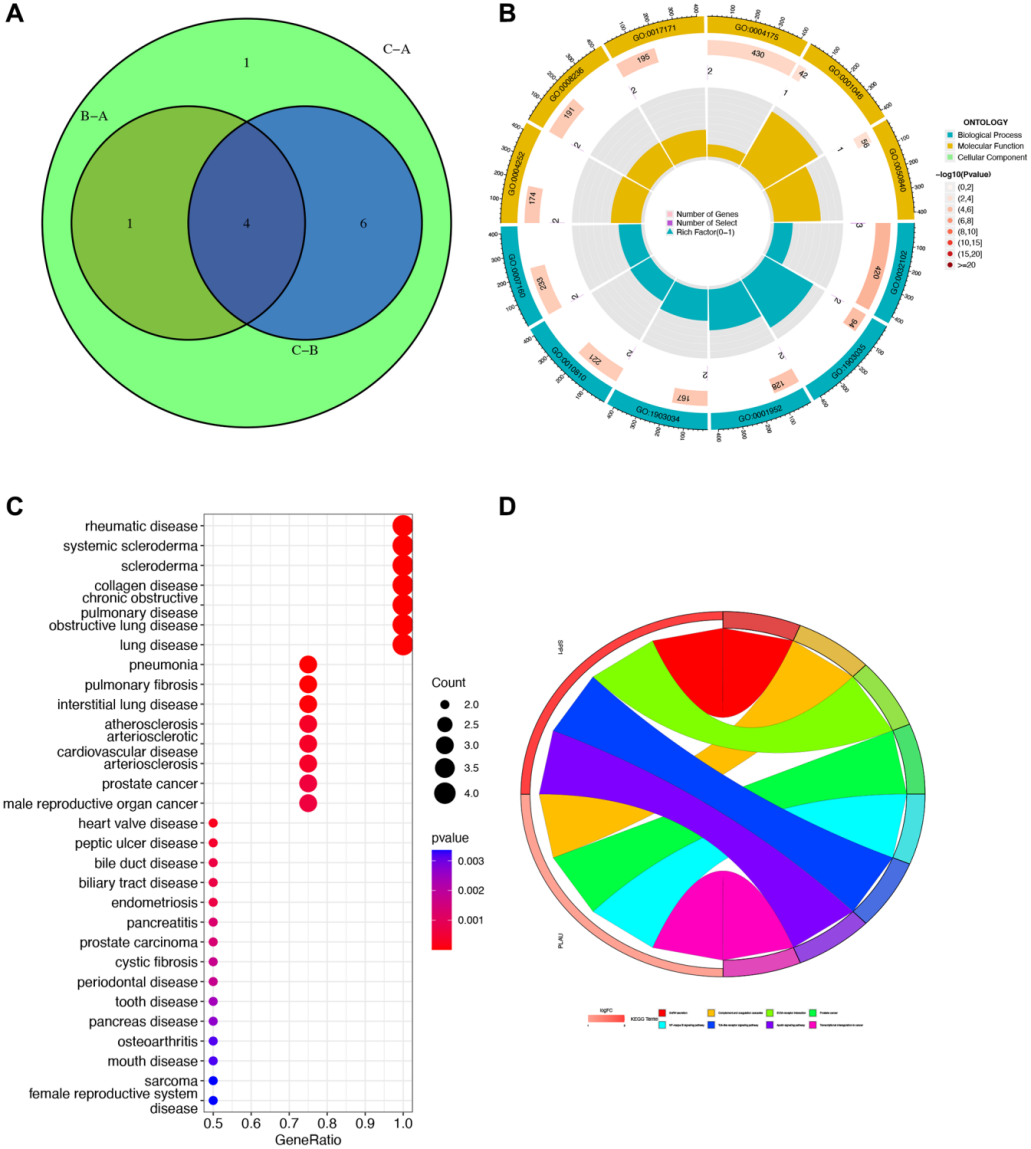
**Gene Ontology (GO), Kyoto Encyclopedia of Genes and Genomes (KEGG), and Disease Ontology (DO) enrichment analyses.** (**A**) Venn diagram for obtaining hub genes by taking the intersection. (**B**) GO enrichment analysis circle showing gene’s biological process, molecular function, and cellular component. (**C**) DO analysis of the relationship between cancers and gene ratio. (**D**) KEGG pathway map. Different colors represented different pathways, and the value of logFC represented the degree of expression.

In the Venn diagram, it is evident that four genes intersect across Clusters A, B, and C. A detailed analysis of these four genes (MMP12, SPP1, PLAU, and TREM1) is then carried out (Figure 4A). To gain a better understanding of the biological behavior and characteristics of each cluster, we perform GO enrichment analysis on the biological processes, molecular functions, and cellular components of these four genes. The analysis reveals their involvement in the negative regulation of responses to external stimuli and activity related to serine-related enzymes (Figure 4B). Furthermore, we explore the relationship between these genes and malignant diseases, revealing strong associations with rheumatic diseases and lung-related diseases (Figure 4C). The subsequent KEGG analysis unveils associations with various pathway disorders and prostate cancer (Figure 4D).

### Construction and inspection of the risk signature

**Figure 5:**
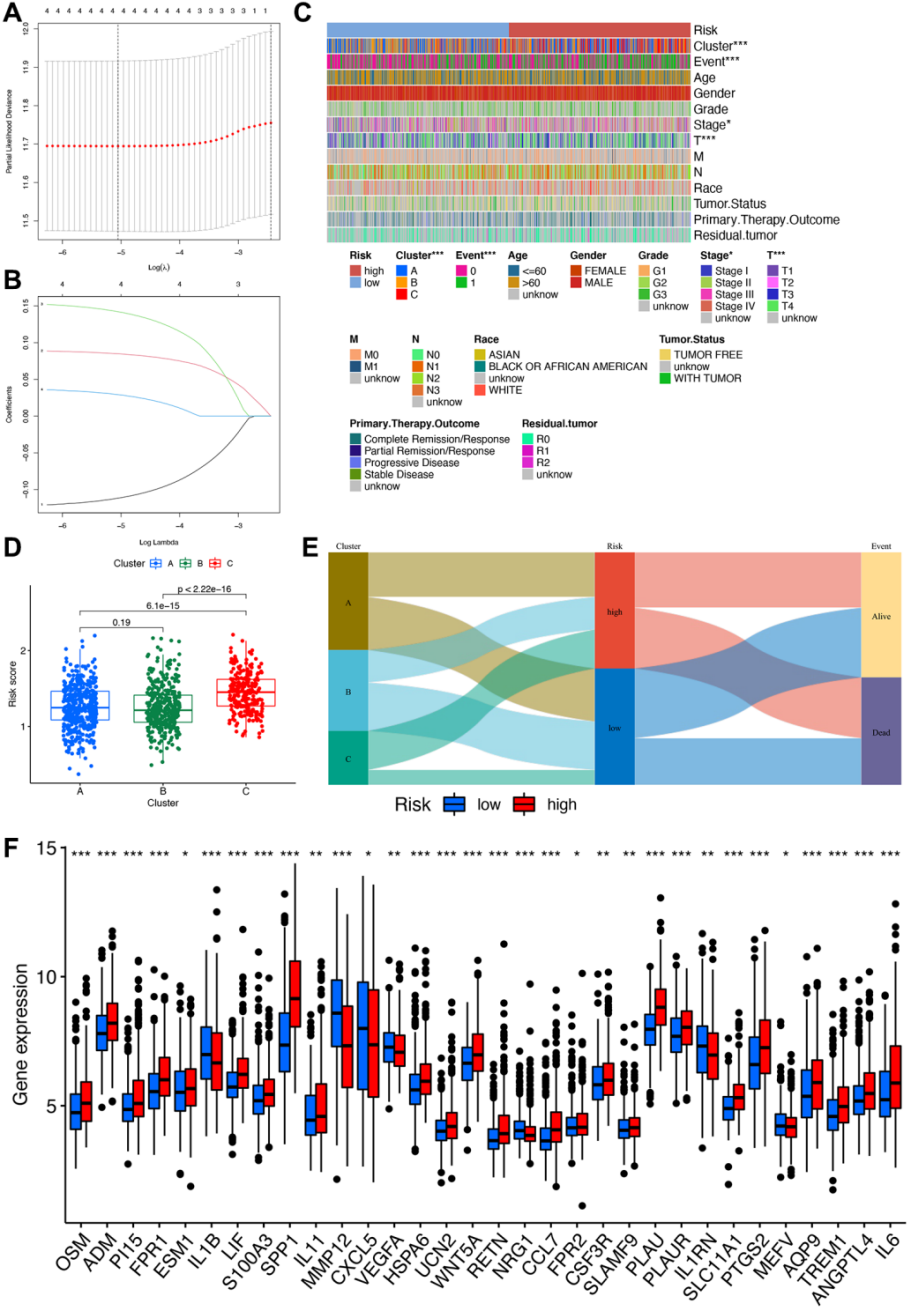
**Construction of the risk signature.** (**A**) Multivariate Cox regression analysis. (**B**) LASSO regression analysis. (**C**) Heatmap of clinical correlations, with asterisks representing differences in this clinical trait between high- and low-risk groups. ^***^p < 0.001; ^**^p < 0.01; ^*^p < 0.05. (**D**) Clinical correlation analysis of differences in risk scores between different clusters. (**E**) Sankey diagram linking cluster, risk, and prognosis. (**F**) Box plot for differential analysis of IRGs in high- and low-risk groups.

Utilizing LASSO and multivariate Cox regression analysis, we identified three hub genes (MMP12, SPP1, PLAU) from the aforementioned four IRGs to establish a risk signature (Figure 5A, 5B). Employing the Caret R package, we divided the total number of STAD patients (*n* = 1043) into two groups: a training group (*n* = 522) and a testing group (*n* = 521), with the former being used for signature development. The risk score was formulated based on the results of the multivariate Cox regression analysis as follows: Risk score = (−0.1217 MMP12 expression) + (0.0959 SPP1 expression) + (0.1746 PLAU expression). Notably, significant differences in risk scores were observed among different gene clusters, categorizing patients with risk scores below the average as low-risk (*n* = 261) and those with scores above the average as high-risk (*n* = 260).

### Association between the risk signature and clinicopathological parameters of STAD

In-depth analysis of the correlations between risk scores, molecular and genetic classifications, prognosis, and clinical aspects was conducted. A heatmap illustrates that gene cluster B is associated with a more favorable prognosis compared to gene clusters A and C (Figure 5C). Applying the scoring system to all STAD samples confirms previous findings, highlighting that patients in gene cluster B exhibit significantly lower risk scores compared to those in gene clusters A and C (Figure 5D). An alluvial graph is generated to depict the distribution of patients across gene clusters A, B, C, risk scores, and future status. This visualization demonstrates that the low-risk group has a higher likelihood of survival (Figure 5E). Furthermore, a comparison of IRG expression between low and high-risk groups is presented to better elucidate the relationship between the risk score and genetic behaviors (Figure 5F).

### The prognostic implications of the IRG-based risk signature

**Figure 6:**
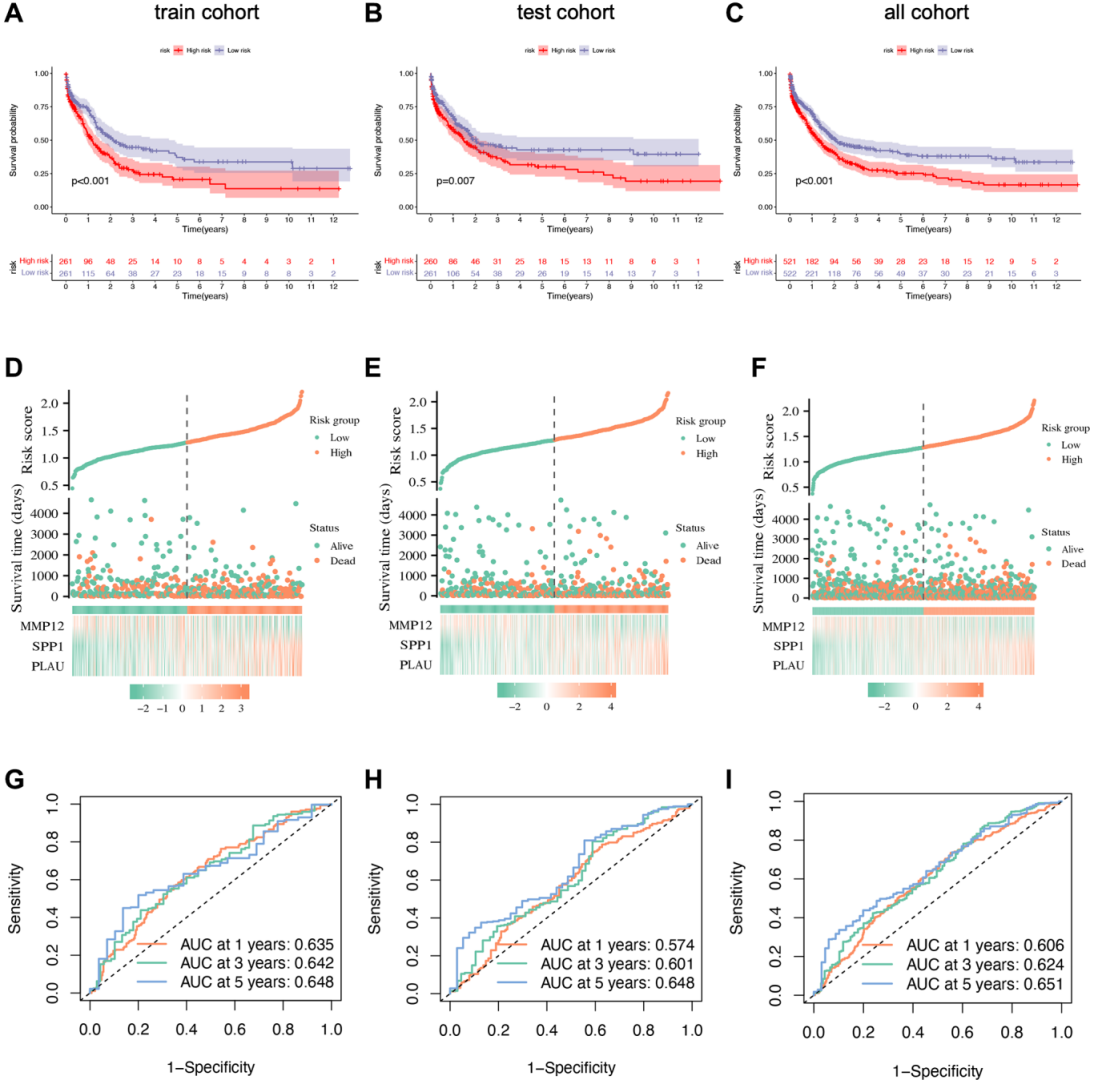
**Prognostic validation of risk signature.** (**A**–**C**) Kaplan–Meier survival analysis with *p*-value < 0.05 indicating that the established risk signature can identify high- and low-risk groups. (**D**–**F**) Scatter plots showing the relationship between risk score and survival time. (**G**–**I**) ROC curve to predict the accuracy of the patient's one-year, three-year, and five-year survival rate.

To validate the prognostic capacity of the IRG-based risk signature, we conducted survival analyses in both the training and testing groups, as well as in the original merged group. The results consistently demonstrate higher survival probabilities for low-risk patients in all three datasets (Figure 6A–6C). Further analysis indicates that favorable IRG expression is more prevalent in the low-risk group, while the opposite holds true for the high-risk group. This highlights the concept that a lower risk score corresponds to a higher likelihood of survival, as confirmed by the risk score distribution plot. By comparing the distributions of the three genes between the low- and high-risk groups, we gain better insight into how these three hub genes influence oncogenesis (Figure 6D–6F). Additionally, ROC curves are generated in the training, testing, and combined groups to further validate the accuracy and reliability of the risk signature (Figure 6G–6I). Furthermore, we assess the risk signature’s ability to predict STAD patients’ future status by analyzing 1-, 3-, and 5-year prognostic classification and prediction efficiency. This analysis reveals significantly higher AUC values.

### External validation of the performance of the IRG-based signature

**Figure 7:**
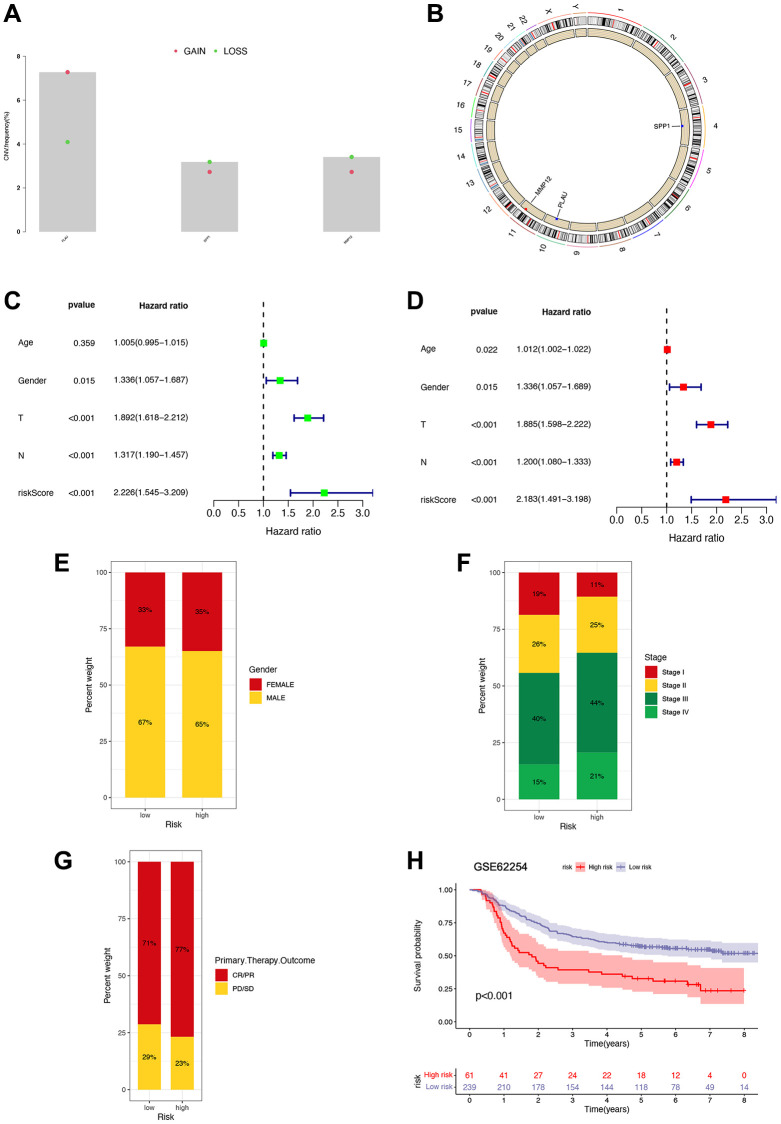
**Prognostic implications of risk signature.** (**A**) Copy number variation frequency analysis with red dots representing copy number gains and green dots representing copy number deletions. (**B**) Copy number circle diagram. The outer circle was the chromosome, and the inner circle was marked with three IRGs. The red dots indicated that the copy number of this gene was increased, and the blue point indicated that the copy number of this gene was more deleted. (**C**, **D**) Univariate and multivariate Cox regression analyses in the combined cohort. (**E**–**G**) The proportion of patients with different gender, stage, and primary therapy outcome in high- and low-risk groups. (**H**) Survival analysis of patients in high- and low-risk groups based on GSE62254 gene.

We also analyzed gene amplification and deletion frequencies for selected genes (Figure 7A). The results indicate a higher frequency of copy number deletions in the SPP1 and MMP12 genes, while the PLAU gene shows more copy number gains. Moreover, we mapped the locations of CNV alterations in genes across the 23 pairs of chromosomes, revealing SPP1 on chromosome 4, PLAU on chromosome 10, and MMP12 on chromosome 11 (Figure 7B). Univariate and multivariate Cox regression analyses conducted on the pooled cohort further confirm the prognostic accuracy of the risk signature (Figure 7C, 7D). In addition, we performed subgroup analyses based on gender, stage, and primary therapy outcomes, suggesting potential correlations between higher risk groups and poorer survival settings (Figure 7E–7G). Survival analysis using the GSE62254 dataset further corroborates our risk signature’s ability to independently predict STAD patients’ prognosis (Figure 7H).

### TME characteristics and potential response to immunotherapy

**Figure 8:**
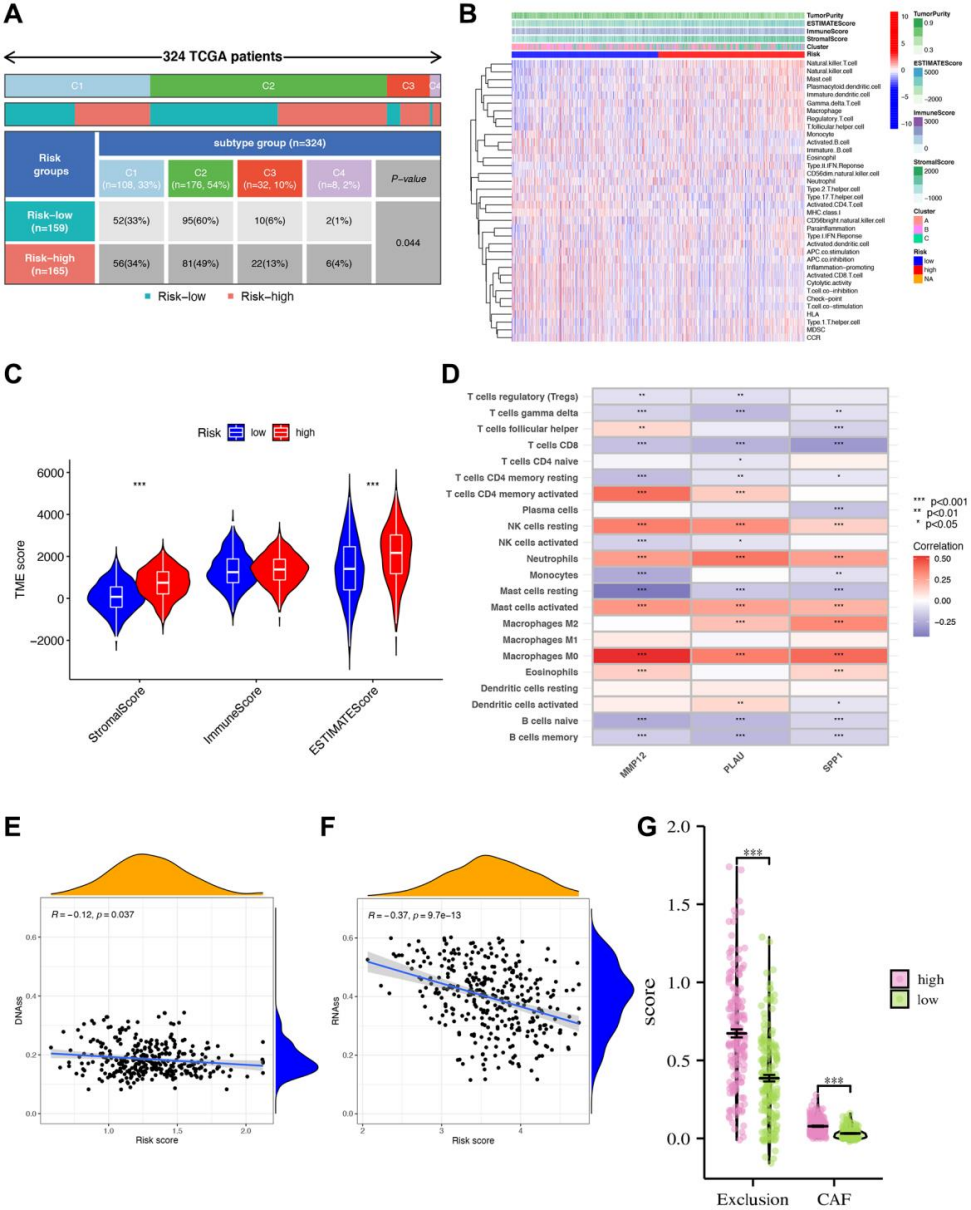
**Tumor microenvironment characteristics (TME) analysis.** (**A**) The clinical correlation analysis showing the number and percentage of patients with each clinical trait in the high- and low-risk groups. (**B**) Immune cell infiltration analysis. (**C**) Analysis of tumor microenvironment differences. ^***^ indicating that tumor microenvironment scores are different between high- and low-risk groups. (**D**) Analysis of immune cell abundance and gene correlation degree. ^***^p < 0.001; ^**^p < 0.01; ^*^p < 0.05. Blue showed a negative correlation, red showed a positive correlation. (**E**, **F**) Risk score and DNA, RNA stemness analysis. Analysis of differences in scores of CAF and other TME populations in high- and low-risk groups. (**G**) Risk score divided differential immune exclusion cells and cancer-associated fibroblasts (CAFs) abundance.

Our analysis of immune cell infiltration in the high- and low-risk groups reveals notable differences (Figure 8A, 8B). Further examination of the TME shows higher stromal cells, immune cells, and comprehensive contents in the high-risk group (Figure 8C). Subsequently, we analyze the relationship between these three IRGs and immune cells using CIBERSORT, finding strong correlations with six immune cell types (macrophages M0, activated mast cells, neutrophils, etc.) (Figure 8D). DNAss and RNAss show negative correlations with the risk score, suggesting that lower risk scores correspond to higher stemness and lower cell differentiation levels (Figure 8E, 8F). In various cell populations, including cancer-associated fibroblasts (CAFs), the high-risk group scores higher than the low-risk group. This could explain the lower survival rate in the high-risk group, resulting from the dynamic interaction of CAFs, tumor-associated fibroblast cells, etc., within the TME, facilitating continuous exchange of nutrients, molecular signals, excretions, etc., (Figure 8G).

**Figure 9:**
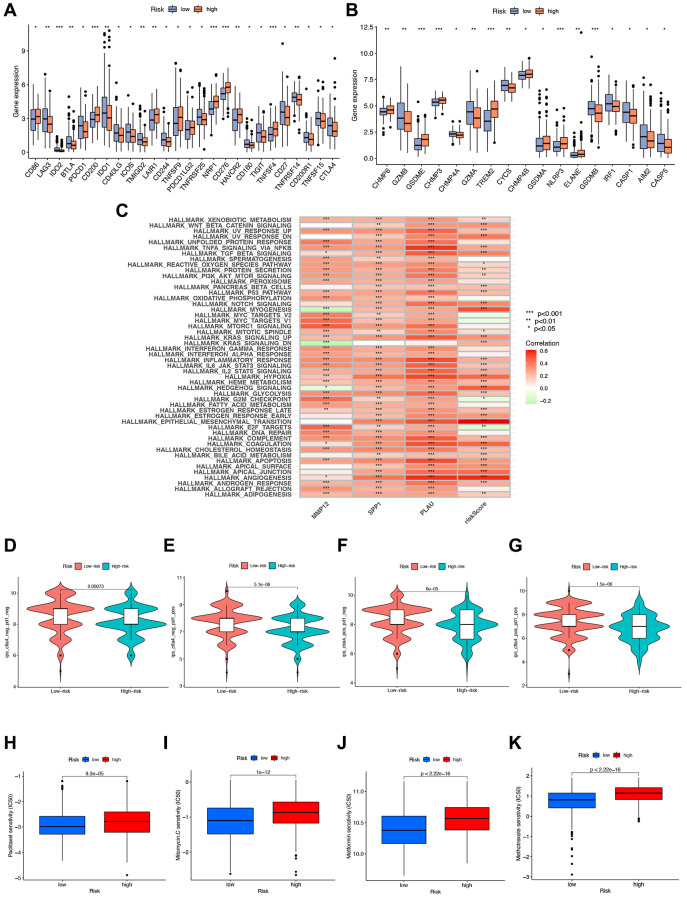
**Immunotherapy analysis.** (**A**, **B**) Gene expression of immune checkpoint molecules and pyroptosis-related genes in high- and low-risk groups. (**C**) GSVA analysis telling which functions or pathways were active in high- and low-risk groups with ^*^ indicated correlation, green indicated negative correlation, and red indicated positive correlation. (**D**–**G**) Immunotherapy analysis to compare the effect of immunotherapy in high- and low-risk groups. (**H**–**K**) Drug sensitivity analysis of various drugs differences between high- and low-risk groups with *p*-value < 0.05 indicating a difference.

We further investigate the correlation between risk signatures and immune cells, pathways, and immune scoring, revealing significant differences in gene expression of immune checkpoint molecules and pyroptosis-related genes (PGs) between high- and low-risk groups (Figure 9A, 9B). Additionally, the three hub IRGs show strong associations with immune pathways (Figure 9C). Furthermore, we evaluate the therapeutic efficacy of immune checkpoint inhibitors (ICIs) using the immunophenotype score (IPS) (Figure 9D–9G). The IPS of the high-risk group is slightly lower than that of the low-risk group under CTLA-4 and PD-1 blockade treatment, indicating that the high-risk group may benefit more from ICB treatment. To enhance STAD patients’ survival rates, we investigate sensitivity differences between high- and low-risk groups, finding that the high-risk group exhibits higher sensitivity to Paclitaxel, Mitomycin C, Metformin, and Methotrexate (Figure 9H–9K).

### Effect of the IRG-based signature on DNA variations landscape

**Figure 10:**
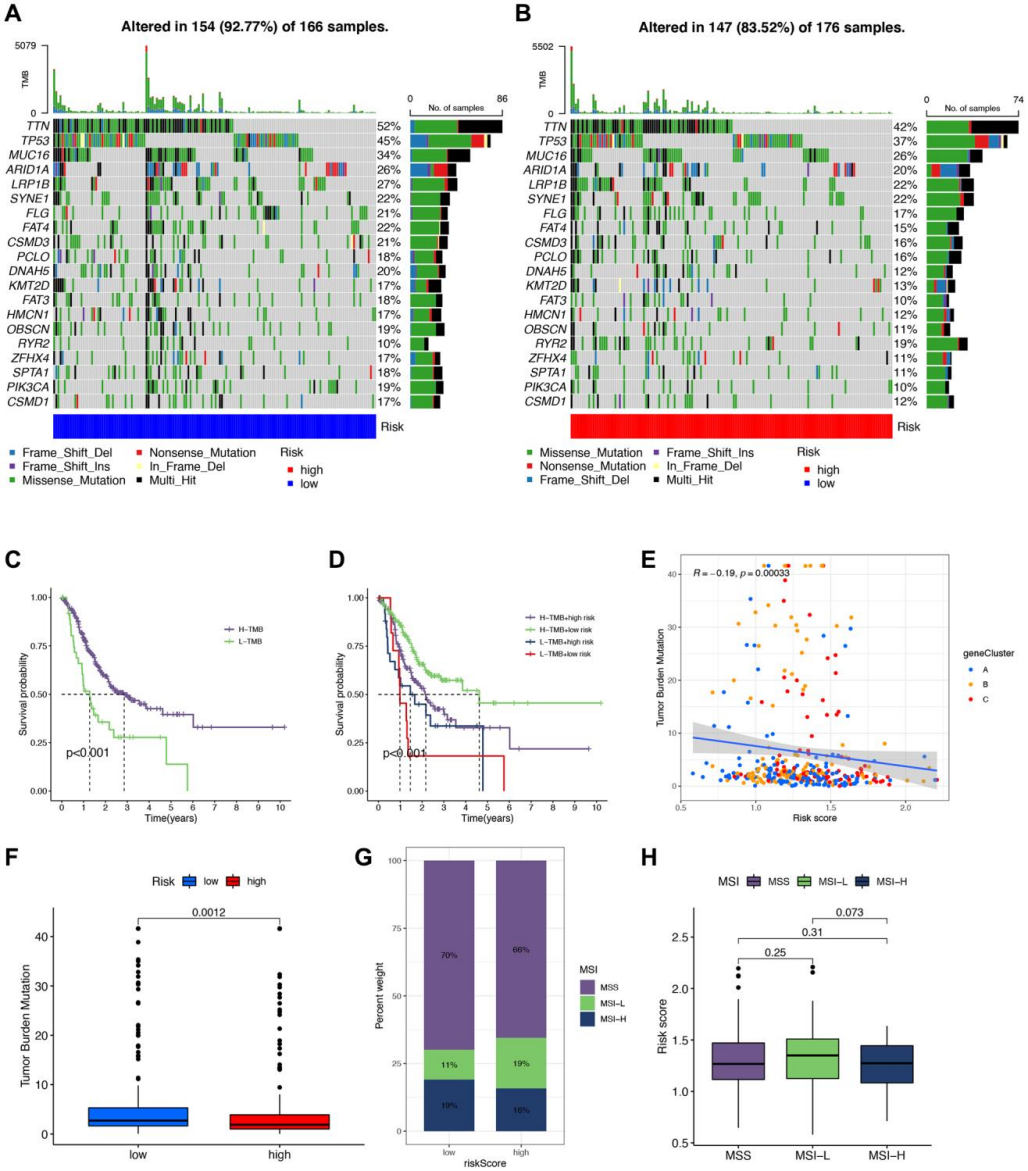
**Correlation between tumor mutational burden (TMB), minicellular instability (MSI), and signature.** (**A**, **B**) Waterfall plots of somatic mutations in low- and high-risk groups, respectively. (**C**, **D**) Survival analysis of different tumor mutational burden (TMB) and risk groups. (**E**) Scatter plot of the association of TMB with gene cluster-based risk scores. (**F**) Analysis of TMB to compare whether there was a difference in TMB of patients in high- and low-risk groups. (**G**, **H**) Correlation analysis of MSI with risk scores.

Additionally, we utilized the maftools software to examine differences in somatic mutation distribution between the low- and high-risk score groups (Figure 10A, 10B). The waterfall plot illustrates the mutations in the top 20 genes across samples and groups with varying TMB and risk scores, allowing for further insights into the relationship between TMB, risk scores, and the prognosis of STAD patients (Figure 10C, 10D). Moreover, we explored the relationship between TMB and risk score using three gene clusters, confirming that a low-risk score corresponds to a high TMB (Figure 10E). TMB quantification analyses validated that the low-risk score group had a higher tumor mutation burden than the high-risk group, aligning with the earlier findings (Figure 10F). Furthermore, we evaluated MSI in the low and high-risk score groups to assess the risk signature’s ability to predict STAD patients’ responsiveness to ICB therapy (Figure 10G). The results indicate a higher proportion of high MSI in the low-risk score group compared to the high-risk score group, suggesting that the former is more sensitive to immunotherapy and has greater therapeutic benefits, further confirming the correlation between the risk signature and MSI (Figure 10H).

### Construction and validation of a nomogram for STAD patients

**Figure 11:**
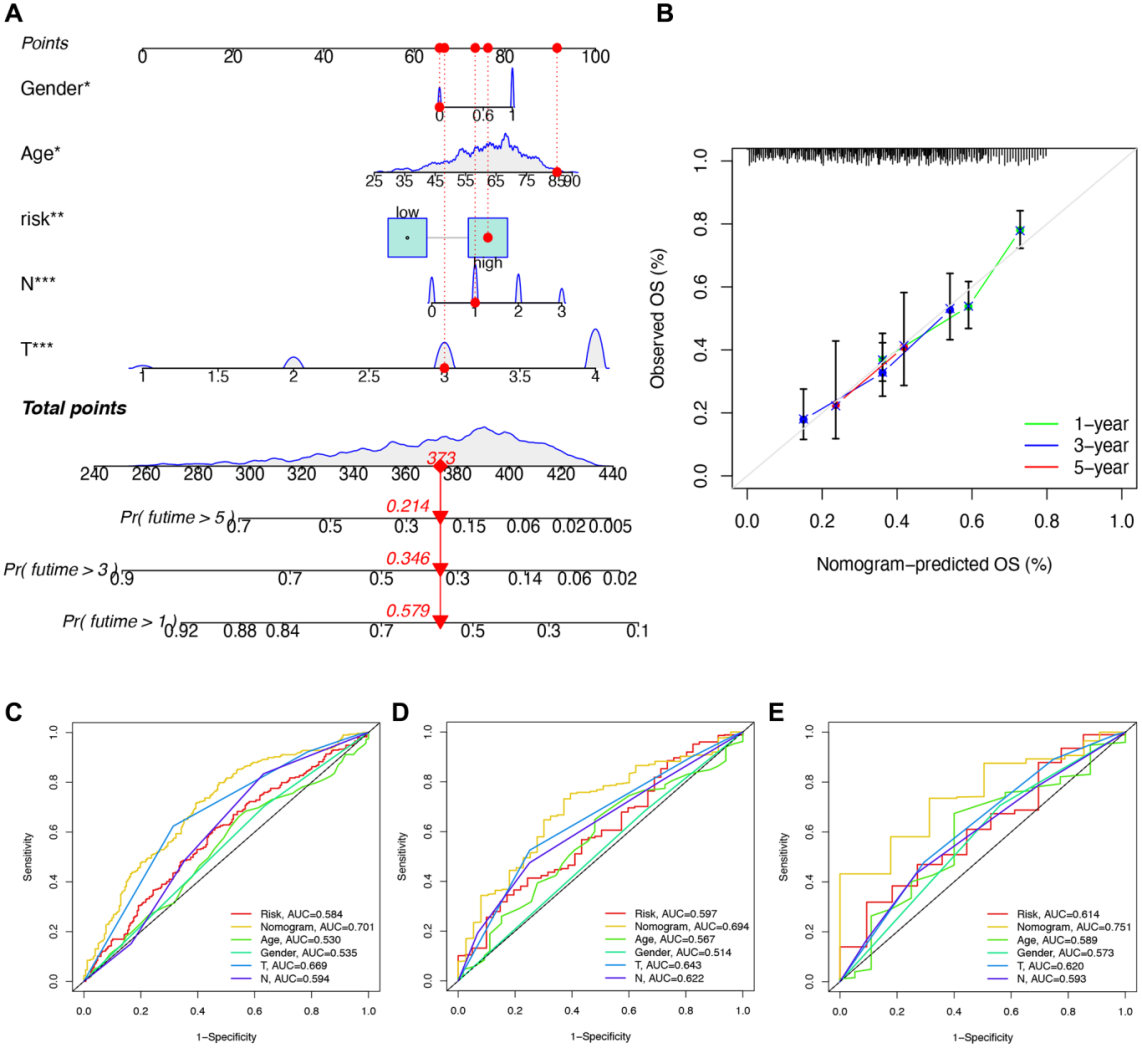
**Construction and validation of a nomogram.** (**A**) Nomogram for getting the score of each clinical trait and calculating the comprehensive score, further predicting the survival of the patient. (**B**) Calibration curve of the nomogram to predict the one-year, three-year, five-year survival rate. (**C**–**E**) ROC curve for validating the accuracy of predicting survival by building the signature.

To predict the 1-, 3-, and 5-year survival rates of STAD patients, we developed a nomogram incorporating the risk score and clinicopathological parameters (Figure 11A). Subsequently, a calibration plot demonstrated that the proposed nomogram performs similarly to the ideal curve (Figure 11B). To bolster the validation, the nomogram displayed the highest AUC values when compared to clinical ROC curves for STAD patients’ 1-, 3-, and 5-year survival, underscoring its superior predictive capabilities (Figure 11C–11E).

### Down-regulation of SPP1 inhibits proliferation and migration abilities of HGC27 and NCI-N87 cells

**Figure 12:**
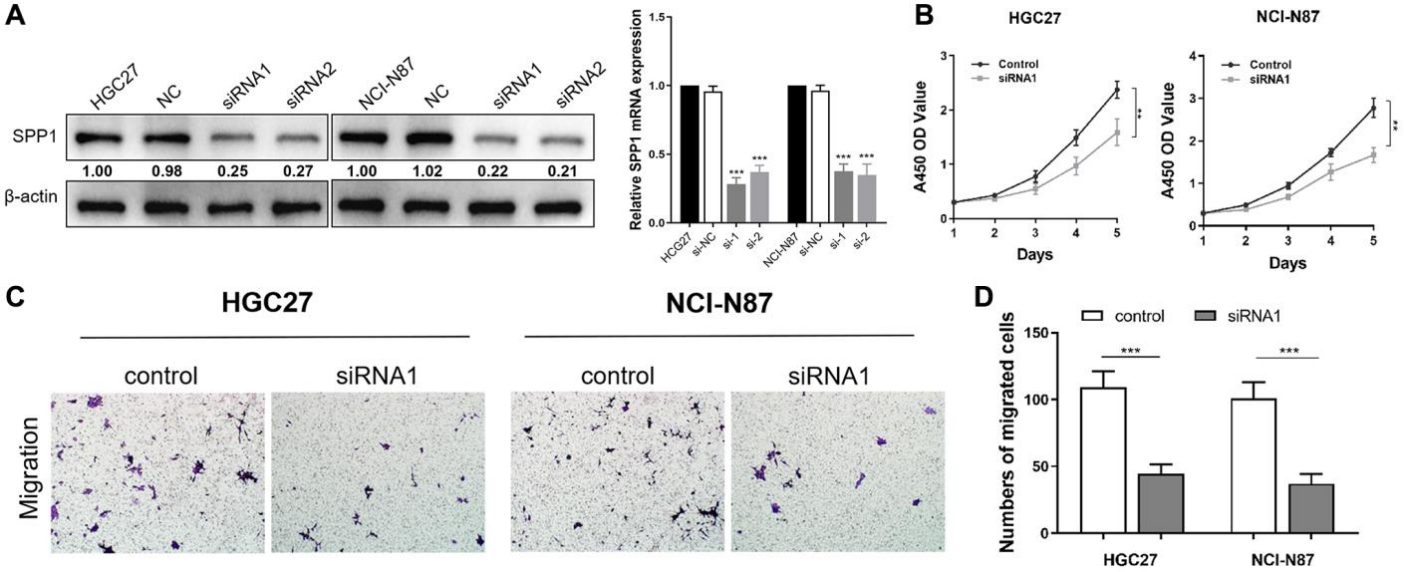
**Down-regulation of SPP1 inhibits proliferation and migration abilities of HGC27 and NCI-N87 cells.** (**A**) Compared with the negative control group in HGC27 and NCI-N87 cells, SPP1 in siRNA transfection group was down-regulated in protein and mRNA expression levels. (**B**) Compared with the control group, the down-regulation of SPP1 significantly inhibited the proliferation of STAD cells. (**C**, **D**) Downregulation of SPP1 expression can significantly inhibit the metastatic ability of gastric cancer cells. ^***^p < 0.001; ^**^p < 0.01.

To gain insights into the malignant behaviors of the hub gene, SPP1, *in vitro*, we validated the down-regulation of SPP1 protein and mRNA expression in siRNA-transfected cells, HGC27, and NCI-N87 using western bloting and RT-qPCR assays (Figure 12A). The CCK-8 assay demonstrated a significant limitation in the proliferative ability of STAD cells when SPP1 expression was down-regulated compared to the control group (Figure 12B). Furthermore, the Transwell cell migration assay indicated that suppressing SPP1 expression markedly restrained the metastatic ability of gastric cancer cells (Figure 12C, 12D). In summary, down-regulating SPP1 significantly suppressed the proliferation and migration capacities of HGC27 and NCI-N87 cells.

## DISCUSSION

Gastric cancer (GC) ranks among the leading causes of cancer-related deaths worldwide, marked by increasing morbidity, poor prognosis, and high mortality rates [14]. While established immunotherapy-related biomarkers like programmed death ligand 1 (PD-L1) expression, tumor mutational burden (TMB), microsatellite instability (MSI), and DNA mismatch repair (MMR) are used to predict immunotherapy efficacy in various malignancies, the precise impact of immune-related genes (IRGs) on the tumor immune microenvironment and the underlying molecular mechanisms in GC remain poorly understood.

To address these knowledge gaps, we analyzed a comprehensive set of 2660 IRGs, ultimately identifying four key genes through rigorous screening. Enrichment analysis shed light on their involvement in negative regulation of responses to external stimuli and activity in serine-related enzymes, which are closely associated with tumor invasion and metastasis. Furthermore, these IRGs demonstrated links to other diseases, such as prostate cancer. Our investigation into immune cell infiltration revealed that the high-risk group exhibited a higher stromal cell content, potentially contributing to disruptions in the adhesion and tissue barriers of normal tissues, thereby promoting rapid tumor development [15–17]. This further validates the role of these four IRGs, particularly in the high-risk group, in driving tumor invasion and metastasis, ultimately diminishing patient survival rates.

As targeted immunotherapy tailored to the tumor microenvironment (TME) remains an evolving field, we delved into the intricate connections between risk signatures, immune cells, pathways, and immune scores. The TME constitutes a complex system comprising diverse cells and cytokines, intimately linked to tumorigenesis, tumor progression, and resistance to immunotherapy [18]. Our immune cell correlation analysis revealed strong associations between three IRGs and six immune cell types, notably Macrophages M0 and Macrophages M2, among others. Macrophages, abundant within the immune cells, exert a pivotal role in tumor progression. Recent research corroborates that both M1 and M2 macrophage phenotypes may foster tumor growth, aligning with our findings [19, 20]. Of note, our study evaluated the effect of immune checkpoint inhibitors (ICIs) via Immunophenotype Score (IPS). The high-risk group displayed lower IPS but greater drug sensitivity, suggesting that despite poorer prognoses in high-risk STAD patients, ICB and drug therapies can effectively manage the disease [21, 22].

Somatic mutations and the tumor immune microenvironment significantly impact GC, influencing tumorigenesis, progression, and drug resistance [23, 24]. TMB serves as a crucial metric for immunotherapy assessment. Interestingly, the low-risk group exhibited higher TMB and MSI, rendering it more sensitive to ICIs, a trend associated with improved survival rates, corroborating previous findings [25]. MSI denotes the phenomenon of microsatellite alleles’ alteration during DNA replication, fostering genomic instability and elevating tumor susceptibility. Moreover, clinical trials suggest that MSI-high GC patients benefit from more extended survival than MSI-low counterparts, emphasizing the potential of MSI as a predictive and prognostic biomarker [26].

In our study, we identified three pivotal hub genes: MMP12, SPP1, and PLAU. MMP12 expression is notably high in GC tissue, escalating with tumor development and metastasis. Patients with MMP12-positive gastric cancer tend to exhibit worse overall survival compared to MMP12-negative patients [27]. Matrix metalloproteinases (MMPs), including MMP12, dismantle various extracellular matrix (ECM) protein components, dissolving connective tissue between cells and within vascular layers, allowing tumor cells to escape their original location and initiate metastasis [28]. MMPs also influence cell surface bioactive molecules, regulating cells and signaling pathways. Multiple lines of evidence link MMPs to tumor invasion, neoangiogenesis, and metastasis [29]. SPP1, encoding osteopontin, a multifunctional adhesive protein expressed by various tissue cells, plays roles in cellular processes like fusion, migration, and motility [30, 31]. Notably, our study is among the first to identify SPP1’s significant role in GC. Existing research underscores that SPP1 overexpression promotes hepatocellular carcinoma metastasis and ovarian cancer drug resistance [32, 33]. Our study further validated that down-regulating SPP1 markedly curtailed the proliferation and migration capacities of gastric cancer cells, underscoring its potential as a therapeutic target.

PLAU, implicated in blood coagulation, wound healing, and cell-matrix adhesion, demonstrates potential as an effective prognostic biomarker and therapeutic target for GC [34]. Research in head and neck squamous cell carcinoma (HNSCC) highlights PLAU’s involvement in cell-matrix adhesion, tissue migration, and extracellular matrix binding, facilitating the epithelial stromal transformation (EMT) process and affecting prognosis [35]. In summary, higher MMP12, SPP1, and PLAU expression in the high-risk group is associated with lower survival rates.

The study of gastric cancer (GC) presents significant challenges due to its unclear molecular pathogenesis and persistently poor prognosis. Consequently, surgical intervention remains the primary treatment modality for GC [23]. Various efforts have been dedicated to constructing prognostic signatures by exploring multiple aspects of GC’s pathogenic mechanisms, aiming to enhance prognostic assessment and contribute to therapeutic advancements. For instance, Ren et al. developed an immune-related signature consisting of four genes (MAGED1, ACKR3, FZD2, and CTLA4), highlighting the promising performance of immune-related signatures in GC treatment and prognosis prediction. This signature, however, was based on publicly available datasets and has yet to be validated in clinical cohorts. There is a pressing need for prospective clinical studies that bridge the gap between immunotherapy and fundamental research [36]. Another predictive signature relying on six immune risk genes (BRD8, CCL25, CMTM3, FPR1, GDF10, and LEPR), closely linked to survival, provided insights into the intersection of chemotherapy, chemoradiotherapy, and the immune system. Nevertheless, it lacked investigations into mutations specific to STAD [37]. Li et al. introduced a prognostic risk score signature comprised of nine differentially expressed IRGs (RBP7, DES, CCR1, PNOC, SPP1, VIP, TNFRSF12A, TUBB3, and PRKCG). This signature illuminated the biological roles of these IRGs in GC and identified novel gene targets for GC treatment. However, it didn’t delve deeply into the tumor microenvironment [38]. Shao et al. constructed a prognostic signature centered on ten ferroptosis-related genes (SP1, MYB, ALDH3A2, KEAP1, AIFM2, ITGB4, TGFBR1, MAP1LC3B, NOX4, and ZFP36) to evaluate the prognosis and immunotherapy in GC patients. The intricate mechanisms through which these ten iron death-related genes interact to influence tumorigenesis and immune processes remain unclear and warrant further investigation [39]. In comparison, our signature offers a comprehensive assessment by considering the variability and immune attributes of IRGs in STAD patients, demonstrating superior reliability compared to other signatures.

In conclusion, our study unveils a novel risk signature with dual capabilities—it effectively predicts GC patient prognosis while illuminating the immune microenvironment within tumors. This finding holds substantial potential for guiding targeted therapy and personalized immunotherapy, ultimately extending the lifespan of patients diagnosed with gastric cancer.

## CONCLUSION

In addition, our research elucidates the critical role of immune-related signatures in STAD, offering fresh insights into the selection of biomarkers, indicators of disease progression and prognosis, as well as potential immunotherapeutic targets. SPP1 emerges as a notable independent prognostic factor for STAD, potentially regulating its progression by exerting influence over the immune microenvironment.

## Supplementary Materials

Supplementary Table 1
