# Supplementary material for: Immunobiological signatures and the emerging role of SPP1 in predicting tumor heterogeneity, malignancy, and clinical outcomes in stomach adenocarcinoma
Source: Aging (Albany NY). 2023 Oct 26;15(20):11588–610. doi: 10.18632/aging.205148 (PMC10637809; doi:10.18632/aging.205148)
Supplement: Supplementary Table 1 [file aging-15-205148-s001.pdf]

SUPPLEMENTARY TABLE

Supplementary Table 1. 12 genes exhibiting p-values less than 0.05 were selected for further analysis.

ID
MMP12
IL6
PLAU
WNT5A
IL1A
PTGS2
ADM
ESM1
RETN
SPP1
ANGPTL4
TREM1
